# Identification of Potential Molecular Targets and Active Ingredients of Mingmu Dihuang Pill for the Treatment of Diabetic Retinopathy Based on Network Pharmacology

**DOI:** 10.1155/2022/2896185

**Published:** 2022-11-24

**Authors:** Yini Zhou, Gujing Fan, Yadong Zhang, Luning Xu

**Affiliations:** ^1^Department of Ophthalmology, Wellem Medical Group, Shanghai 200135, China; ^2^Department of Ophthalmology, Huangpu Branch, Shanghai Ninth People's Hospital Affiliated to Shanghai Jiaotong University School of Medicine, Shanghai 200011, China; ^3^Department of Endocrinology, Affiliated Sanming First Hospital of Fujian Medical University, Sanming, Fujian Province 365000, China; ^4^Department of Clinical Pharmacy, Affiliated Sanming First Hospital of Fujian Medical University, Sanming, Fujian Province 365000, China

## Abstract

**Objective:**

Mingmu Dihuang Pill (MMDHP) is a traditional Chinese formula that has shown remarkable improvements of dry eyes, tearing, and blurry vision; however, the mechanisms underlying MMDHP treatment for diabetic retinopathy have not been fully understood. This study is aimed at identifying the molecular targets and active ingredients of MMDHP for the treatment of diabetic retinopathy based on network pharmacology.

**Methods:**

All active ingredients of MMDHP were retrieved from TCMSP and BATMAN-TCM databases, and the targets of active ingredients of MMDHP were predicted on the SwissTargetPrediction website. Diabetic retinopathy-related target sets were retrieved from GeneCards and OMIM databases, and the intersecting targets between targets of active ingredients of MMDHP and potential therapeutic targets of diabetic retinopathy were collected to generate the traditional Chinese medicine-ingredient-target-diabetic retinopathy network and to create the protein-protein interaction network. In addition, GO terms and KEGG pathway enrichment analyses were performed to identify the potential pathways, and molecular docking was employed to verify the binding of active ingredients of MMDHP to key targets of diabetic retinopathy.

**Results:**

Network pharmacology predicted 183 active ingredients and 904 targets from MMDHP, and 203 targets were intersected with the therapeutic targets of diabetic retinopathy. The top 10 hub targets included PIK3RA, TP53, SRC, JUN, HRAS, AKT1, VEGFA, EGFR, ESR1, and PI3KCA. GO terms and KEGG pathway enrichment analyses identified AGE-RAGE, PI3K-AKT, and Rap1 signaling pathways as major pathways involved in MMDHP treatment for diabetic retinopathy. Molecular docking confirmed a good binding affinity of active ingredients of MMDHP, including luteolin, acacetin, naringenin, and alisol B, with AKT1, SRC, and VEGFA as the three key targets of diabetic retinopathy.

**Conclusion:**

MMDHP may be effective for the treatment of diabetic retinopathy through active ingredients luteolin, acacetin, naringenin, and alisol B via AKT1, SRC, and VEGFA in AGE-RAGE, PI3K-AKT, and Rap1 signaling pathways.

## 1. Introduction

Diabetes mellitus, a group of metabolic diseases characterized by chronic hyperglycemia, is a major health problem worldwide, which greatly threatens human health and global economy [1]. The global number of people living with diabetes and global health expenditures due to diabetes were estimated to be 536.6 million USD and 966 billion USD in 2021, and these figures were projected to be 783.2 million USD and 1,054 billion USD in 2045 [2]. As one of the leading causes of mortality and disability worldwide [3], diabetes was the 9th leading cause of mortality worldwide and approximately 1.5 million people died of this chronic metabolic disorder in 2019 [4], while the death was predicted to increase to 1.59 million using an autoregressive integrated moving average (ARIMA) model in 2025 [5].

Diabetic retinopathy, one of the most common and most severe complications of diabetes, is a chronic, progressive microvascular disorder that may lead to vision-threatening damages to the retina and even blindness [6]. There are two types of diabetic retinopathy, including nonproliferative diabetic retinopathy (NPDR) at the early stage, which is characterized by increased vascular permeability, retinal hemorrhage, and pericyte exfoliation from retinal vessels; and proliferative diabetic retinopathy (PDR) at the late stage, which is characterized by pathologic neovascularization, retinal scarring, and detachment and vitreous hemorrhage [6]. A recent meta-analysis showed a 22.27% (95% confidential interval (CI): 19.73% to 25.03%) global prevalence rate of diabetic retinopathy among individuals living with diabetes mellitus and 103.12 million adults with diabetic retinopathy in 2020 and 160.5 million adults were predicted to have with diabetic retinopathy in 2045 [7]. In China, the prevalence of diabetic retinopathy was estimated to be 1.14% among general populations and 18.45% among individuals with diabetes mellitus [8]. These data imply the urgent need of interventions targeting diabetic retinopathy.

Mingmu Dihuang pill, a traditional Chinese formula that consists of multiple medicinal plants in optimal prescriptions, including *Chrysanthemum morifolium*, *Lycium barbarum*, *Moutan cortex*, *Dioscoreae rhizoma*, *Poria cocos*, *Alisma orientalis*, *Cornus officinalis*, *Paeonia lactiflora*, *Tribulus terrestris*_,_ and *Concha haliotidis* [9], has shown remarkable improvements of dry eyes, tearing, and blurry vision [10]. Previous studies have demonstrated that Mingmu Dihuang pills are effective to reduce the area of ecchymosis and number of retinal microangioma and to improve the vision sight among patients with diabetic retinopathy [10–14]. However, the targets and active ingredients of Mingmu Dihuang pill for the treatment of diabetic retinopathy have not been fully understood until now [15].

**Figure 1:**
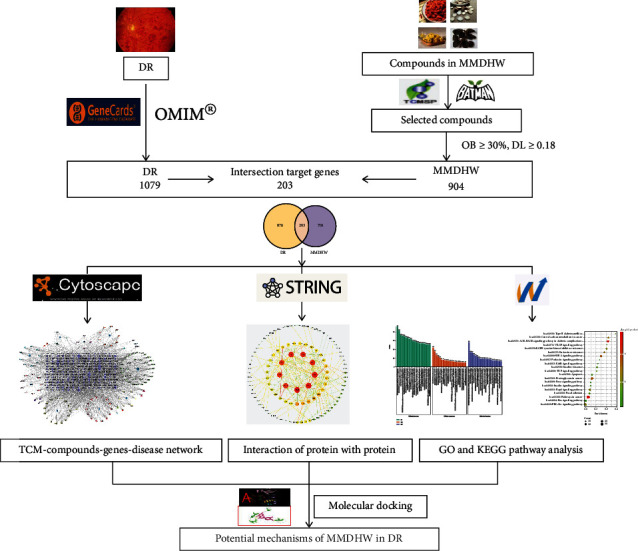
Flow chart of the study.

In the present study, the active ingredients and potential molecular targets of Mingmu Dihuang pill for the treatment of diabetic retinopathy were screened using network pharmacology, and the key targets and signaling pathways involved in Mingmu Dihuang pill treatment for diabetic retinopathy were identified and verified using molecular docking [16–20] (Figure 1). Our findings may provide insights into the development of novel agents for the treatment for diabetic retinopathy.

## 2. Methods

### 2.1. Screening of Active Ingredients of Mingmu Dihuang Pill

All active ingredients of Mingmu Dihuang pills were retrieved from the Traditional Chinese Medicine Systems Pharmacology Database and Analysis Platform (TCMSP) (https://tcmsp-e.com/) and the Bioinformatics Analysis Tool for Molecular mechANism of Traditional Chinese Medicine (BATMAN-TCM) database (http://bionet.ncpsb.org.cn/batman-tcm/), including *Chrysanthemum morifolium*, *Lycium barbarum*, *Moutan cortex*, *Dioscoreae rhizoma*, *Poria cocos*, *Alisma orientalis*, *Cornus officinalis*, *Paeonia lactiflora*, *Radix rehmanniae preparata*, *Angelica sinensis*, *Tribulus terrestris*, and *Concha haliotidis*. Active ingredients with oral bioavailability (OB) of 30% and higher and drug likeness (DL) of 0.18 and greater were screened.

### 2.2. Prediction of Targets of Active Ingredients of Mingmu Dihuang Pill

To predict the targets of active ingredients of Mingmu Dihuang pill, the sdf files regarding the active ingredients of Mingmu Dihuang pill were downloaded from the PubChem database (https://pubchem.ncbi.nlm.nih.gov) or the mol2 files were downloaded and converted into smiles files using the software Open Babel. Then, the sdf or smiles files were loaded into the SwissTargetPrediction web server (http://swisstargetprediction.ch/). The targets of active ingredients acquired from the SwissTargetPrediction web server with a probability of 0 and higher were selected as potential targets.

### 2.3. Prediction of Targets for Diabetic Retinopathy

Diabetic retinopathy-related genes were retrieved from the GeneCards (https://www.genecards.org) and Online Mendelian Inheritance in Man (OMIM) databases (http://www.omim.org) using the term “diabetic retinopathy”.

### 2.4. Generation of the Traditional Chinese Medicine-Ingredient-Target-Diabetic Retinopathy Network

Diabetic retinopathy-related genes and all targets of active ingredients of Mingmu Dihuang pill were input into the WeiShengxin database (http://bioinformatics.cn), in order to screen the intersection between the diabetic retinopathy-related gene set and the drug target set, and the Venn diagram was plotted. The names of intersecting targets and names and ingredients of Mingmu Dihuang pill were loaded into the network visualization platform Cytoscape version 3.2.1, in order to generate the traditional Chinese medicine-ingredient-target-diabetic retinopathy network.

### 2.5. Creation of a Protein-Protein Interaction (PPI) Network

To create a PPI network, the intersecting targets of Mingmu Dihuang pill and diabetic retinopathy were input into the STRING 11.5 platform (https://string-db.org/cgi/), and the protein type was defined as “*Homo sapiens*” using the multiple protein tool and was saved as a TSV-format file. The PPI network was then visualized using the software Cytoscape version 3.2.1.

### 2.6. Gene Ontology (GO) Terms and Kyoto Encyclopedia of Genes and Genomes (KEGG) Pathway Enrichment Analyses

GO terms and KEGG pathway enrichment analyses were performed to investigate the biological processes and metabolic pathways of all intersecting targets between Mingmu Dihuang pill and diabetic retinopathy, using the online Metascape platform (http://metascape.org/gp/index.html).

### 2.7. Molecular Docking Verification

According to the degree values of active ingredients in the traditional Chinese medicine-ingredient-target-diabetic retinopathy network, the degree value of the node in the PPI network and GO terms and KEGG pathway enrichment analysis results, four major active ingredients (alisol B, luteolin, naringenin, and acacetin) and three key targets AKT1 (PDB ID: 4EJN), SRC (PDB ID: 4U5J), and VEGFA (PDB ID: 3BDY) were selected for molecular docking. The AKT1 protein was fixed at the grid center set to 32, 42, and 13 for x, y, and z and at the grid box size set at 64, 64, and 64 Å for x, y, and z, respectively, and the SRC protein was fixed at the grid center set to −3.1, 51, and 25 for x, y, and z and at the grid box size set at 90, 77, and 104 Å for x, y, and z, respectively, while the VEGFA protein was fixed at the grid center set to −39, −57, and −3 for x, y, and z and at the grid box size set at 65, 138, and −365 Å for x, y, and z, respectively. The grid box size was set using the software AutoDock Vina version 1.5.6, and the docking results were visualized using the PyMOL software [21]. A negative binding energy indicates a high likelihood of the binding between the ligand and receptor [15, 22, 23].

## 3. Results

### 3.1. Active Ingredients and Target Genes of Mingmu Dihuang Pill

A total of 183 active ingredients of Mingmu Dihuang pill were retrieved in the TCMSP database, including 20 active ingredients of *C. morifolium*, 45 active ingredients of *L. barbarum*, 2 active ingredients of *R. rehmanniae preparata*, 11 active ingredients of *M. cortex*, 16 active ingredients of *D. rhizoma*, 15 active ingredients of *P. cocos*, 10 active ingredients of *A. orientalis*, 20 active ingredients of *C. officinalis*, 2 active ingredients of *A. sinensis*, 13 active ingredients of *P. lactiflora*, 12 active ingredients of *T. terrestris*, and 4 active ingredients of *C. haliotidis*. Following removal of the active ingredients with unidentified targets, the active ingredients of Mingmu Dihuang pill were finally predicted (Supplementary Table 1). A total of 13 common ingredients were identified, including *C. morifolium* and *T. terrestris* (A); *C. morifolium*, *M. cortex*, *P. lactiflora*, and *T. terrestris* (B); *C. morifolium*, *L. barbarum*, and *M. cortex* (C1); *C. morifolium*, *L. barbarum*, *R. rehmanniae preparata*, *C. officinalis*, *A. sinensis*, and *P. lactiflora* (C2); *C. morifolium* and *C. officinalis* (D); *L. barbarum* and *C. officinalis* (E1); *L. barbarum* and *C. officinalis* (E2); *L. barbarum*, *C. officinalis*, *R. rehmanniae preparata*, *D. rhizoma*, and *A. sinensis* (E3); *L. barbarum* and *D. rhizome* (F1); and *L. barbarum* and *D. rhizome* (F2); the common ingredients of G1 include *M. cortex* and *P. lactiflora* (G1); *M. cortex* and *P. lactiflora* (G2); and *M. cortex*, *A. orientalis*, *C. officinalis*, *P. lactiflora*, and *T. terrestris* (G3). Then, the targets of active ingredients of Mingmu Dihuang pill were predicted in the SwissTargetPrediction platform and 402 targets of *C. morifolium* actions, 390 targets of *L. barbarum* actions, 44 targets of *R. rehmanniae preparata* actions, 221 targets of *M. cortex* actions, 345 targets of *D. rhizoma* actions, 284 targets of *P. cocos* actions, 301 targets of *A. orientalis* actions, 313 targets of *C. officinalis* actions, 43 targets of *A. sinensis* actions, 274 targets of *P. lactiflora* actions, 358 targets of *T. terrestris* actions, and 197 targets of *C. haliotidis* actions were identified. Following removal of repeated targets, a total of 904 targets of Mingmu Dihuang pill actions were finally identified.

### 3.2. Common Targets between Diabetic Retinopathy Treatment and Mingmu Dihuang Pill Actions

**Figure 2:**
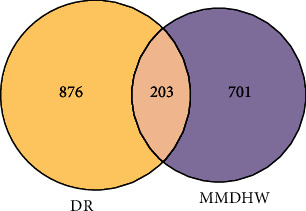
Venn diagram for the intersection between the Mingmu Dihuang pill target set and the diabetic retinopathy-related target set. DR: diabetic retinopathy-related target set; MMDHW: Mingmu Dihuang pill target set.

**Figure 3:**
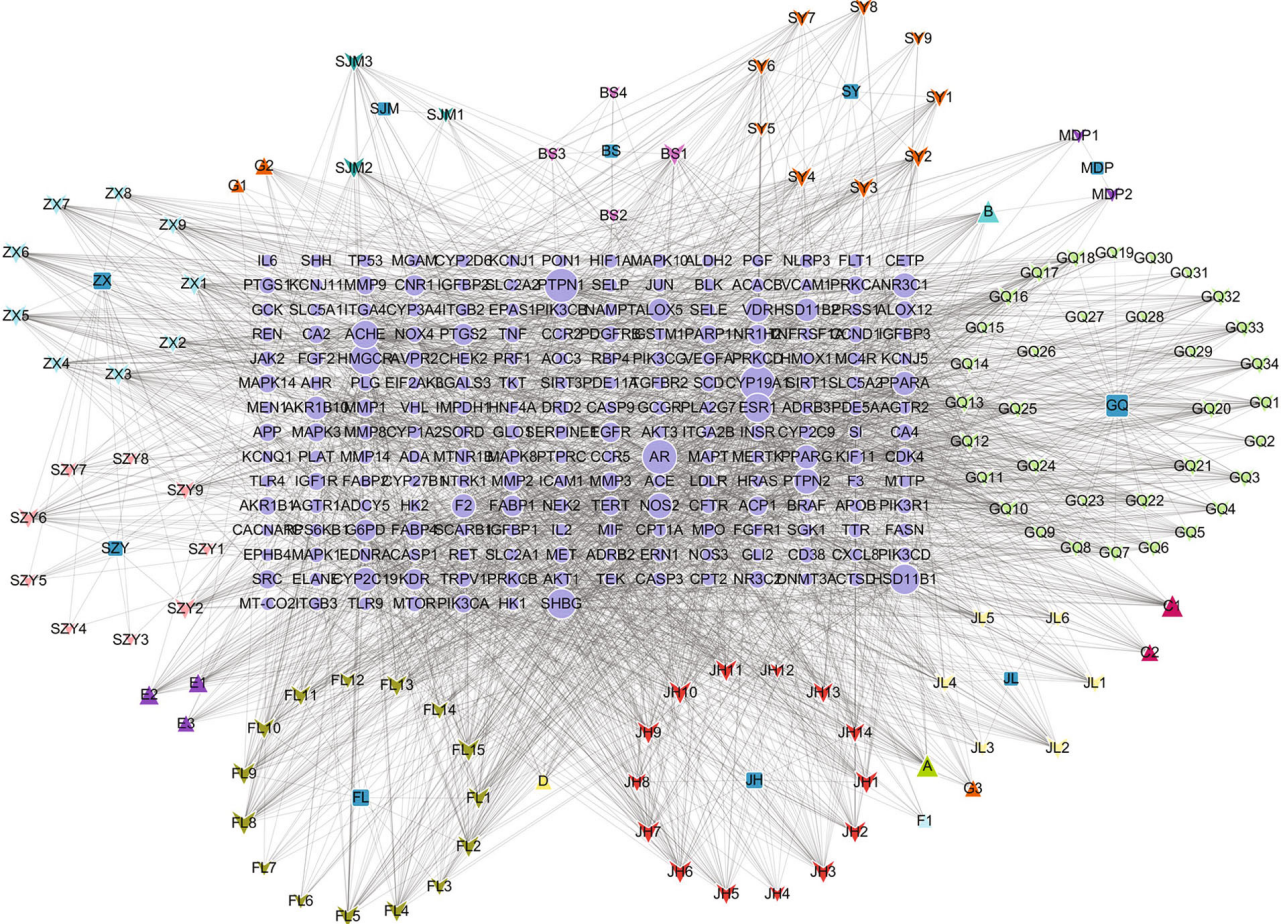
The Mingmu Dihuang pill-ingredient-target-diabetic retinopathy network.

A total of 1,021 diabetic retinopathy-associated target genes were retrieved in the GeneCards database, and 59 target genes were retrieved in the OMIM database. Following target gene merging and removal of repeated targets, a total of 1,079 diabetic retinopathy-associated target genes were identified. Then, these targets were intersected with 904 potential targets of Mingmu Dihuang pill, and 203 potential targets were finally yielded (Figure 2). The traditional Chinese medicine components of Mingmu Dihuang pill and their corresponding active ingredients and potential targets of diabetic retinopathy were input into the Cytoscape software, so as to generate a traditional Chinese medicine-ingredient-target-diabetic retinopathy network (Figure 3). Network topology analysis showed that the seven chemicals with the highest node degree included luteolin, alisol B, acacetin, naringenin, chryseriol, isorhamnetin, and kaempferol, with degrees of freedom of 36, 36, 35, 35, 34, 34, and 34, respectively.

### 3.3. Topology Analysis of the PPI Network

**Figure 4:**
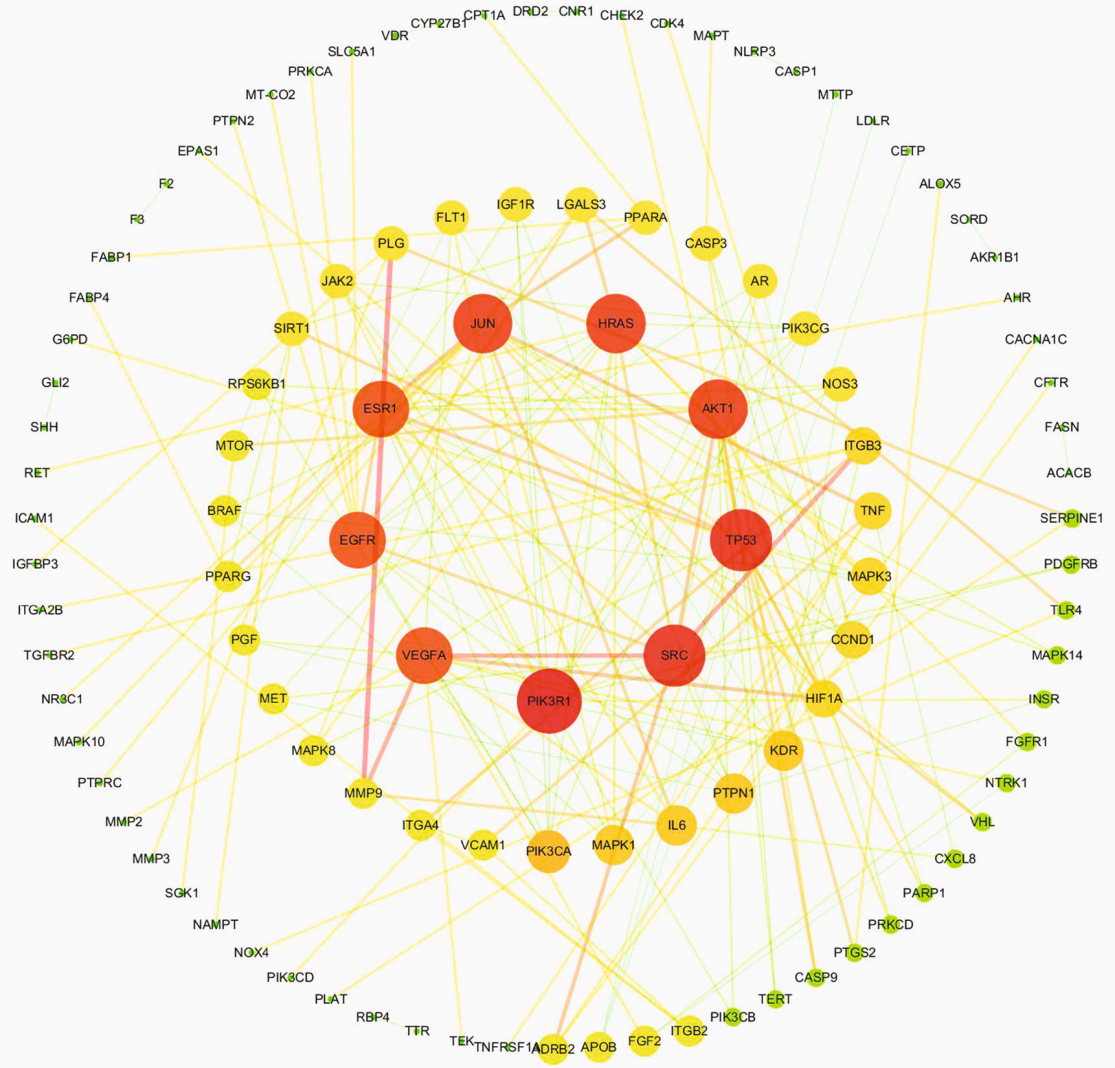
A protein-protein interaction network displays the targets of Mingmu Dihuang pill for treatment of diabetic retinopathy.

The 203 predicted potential targets were input into the STRING platform to generate a PPI network, which contained 203 nodes and 1,169 lines. In this study, the targets with a confidence score of higher than the median were defined as the core targets of Mingmu Dihuang pill, and following screening of the median three times, the targets with a confidence score of > 0.979, 5 were identified as core targets of Mingmu Dihuang pill. The screened targets were visualized using the Cytoscape software and subjected to topology analysis, and a PPI network, which contained 111 nodes and 171 lines, was generated (Figure 4). The top 10 hub proteins included PIK3RA, TP53, SRC, JUN, HRAS, AKT1, VEGFA, EGFR, ESR1, and PI3KCA.

### 3.4. GO Terms Enrichment Analysis Identifies Potential Target Genes of Mingmu Dihuang Pill for Diabetic Retinopathy

**Figure 5:**
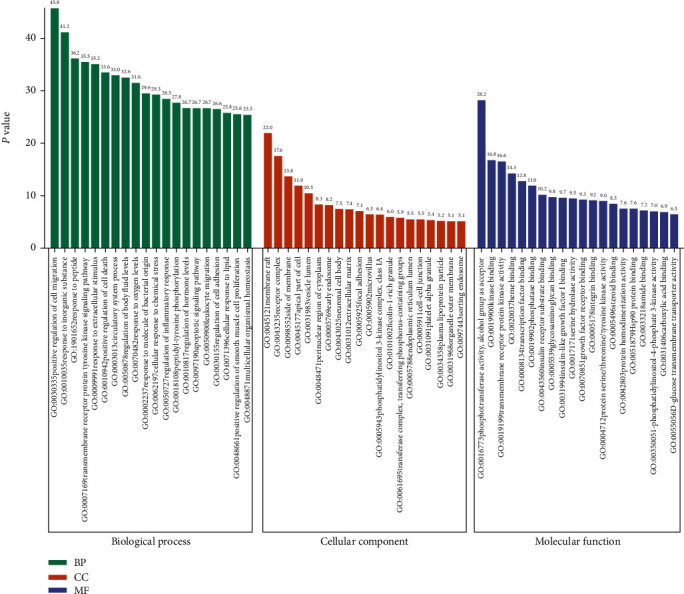
GO enrichment analysis of potential targets of Mingmu Dihuang pill for treatment of diabetic retinopathy. BP: biological process; CC: cellular component; MF: molecular function.

GO annotations of the 203 predicted targets were classified into three categories, including biological processes, molecular functions, and cellular components (Table 1). The target genes relating to biological processes were most significantly enriched in positive regulation of cell migration (GO ID: 0030335), response to inorganic substance (GO ID: 0010035), response to peptide (GO ID: 1901652), transmembrane receptor protein tyrosine kinase signaling pathway (GO ID: 0007169), and response to extracellular stimulus (GO ID: 0009991), and the target genes relating to cellular components were most significantly enriched in membrane raft (GO ID: 0045121), receptor complex (GO ID: 0043235), side of membrane (GO ID: 0098552), apical part of cell (GO ID: 0045177), and vesicle lumen (GO ID: 0031983), while the target genes relating to molecular functions were most significantly enriched in phosphotransferase activity, alcohol group as acceptor (GO ID: 0016773), kinase binding (GO ID: 0019900), transmembrane receptor protein kinase activity (GO ID: 0019199), heme binding (GO ID: 0020037), transcription factor binding (GO ID: 0008134), and phosphatase binding (GO ID: 0019902) (Figure 5).

### 3.5. KEGG Pathway Enrichment Analysis of Potential Target Genes of Mingmu Dihuang Pill for Diabetic Retinopathy

**Figure 6:**
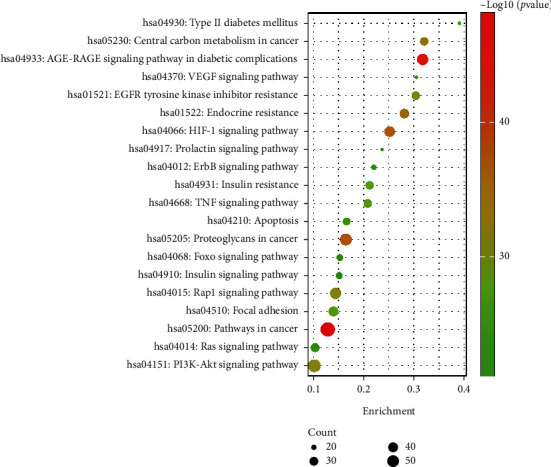
KEGG enrichment analysis of potential targets of Mingmu Dihuang pill for treatment of diabetic retinopathy.

A total of 378 pathways were identified (Table 2). The top 20 hub pathways relating to diabetic retinopathy are shown in Table 3, and Figure 6 displays the bubble plot of these 20 hub pathways. KEGG pathway enrichment analysis revealed that these hub pathways included “pathways in cancer” (hsa05200), “AGE-RAGE signaling pathway in diabetic complications” (hsa04933), “PI3K-AKT signaling pathway” (hsa04151), “Rap1 signaling pathway” (hsa04015), “HIF-1 signaling pathway” (hsa04066), and “endocrine resistance” (hsa01522).

### 3.6. Molecular Docking of Potential Active Compounds with Key Targets of Diabetic Retinopathy

**Figure 7:**
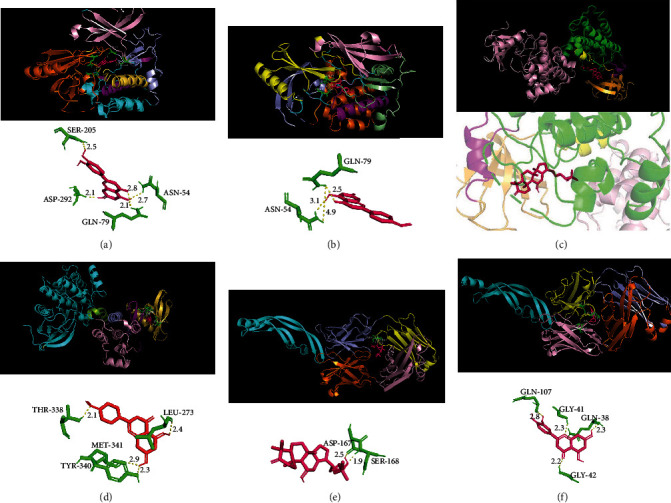
Molecular docking of active compounds of Mingmu Dihuang pill with AKT1, SRC, and VEGFA proteins. The red indicates small-molecule ligands and the green indicates amino acid residues of receptors. (a) AKT1-luteolin; (b) AKT1-acacetin; (c) SRC-alisol B; (d) SRC-acacetin; (e) VEGFA-alisol B; (f) VEGFA-luteolin.

AKT1, SRC, and VEGFA were found to have the binding energy of < −5 kcal/mol with all key targets of diabetic retinopathy (Table 4), suggesting the good binding energy towards the key targets of diabetic retinopathy. Next, molecular docking results with the good binding energy were visualized (Figure 7). Luteolin was found to bind to Ser205, Asp292, Gln79, and Asn54 of AKT1, which increased the structural stability and had good receptor-ligand binding energy, and acacetin bound to Asn54 and Gln79 amino acid residues of AKT1, while alisol B was embedded in the SRC protein cavity to form a stable structure. In addition, acacetin interacted with Tyr340, Met341, Leu273, and Thr338 of the SRC protein, which had a high binding affinity and stable binding mode, and alisol B bound to Ser168 and Asp167 of the VEGFA protein, while acacetin interacted with Gln107, Gly41, Gln38, and Gly42 of the VEGFA protein.

## 4. Discussion

Mingmu Dihuang pill, a traditional Chinese formula based on Liuwei Dihuang pill with addition of *C. morifolium*, *L. barbarum*, *Catsia tora*, *P. lactiflora*, *A. sinensis*, and *T. terrestris* in optimal prescriptions, has been used for the clinical treatment of eye dryness, photophobia, blurry eyes, and tears in wind [9, 10]. In rats with diabetic retinopathy, Mingmu Dihuang pill was found to protect the injured retina and improve retinal lesions through promoting the production of autophagosome in retinal tissues, improving retinal microcirculation, and promoting blood flow [11]. In addition, Mingmu Dihuang pill was reported to increase the antioxidative damages in the retina and effectively reduce the damages of oxidative stress to retinal cells in diabetic rats, thereby protecting retinal injury [24]. However, the active ingredients and targets of actions of Mingmu Dihuang pills have not been fully understood.

In this study, we identified 183 active ingredients, 904 targets of Mingmu Dihuang pill, and 203 targets that were intersected with the therapeutic targets of diabetic retinopathy using network pharmacology and molecular docking. PPI network analysis identified 111 hub targets, and the top 10 hub targets included PIK3RA, TP53, SRC, JUN, HRAS, AKT1, VEGFA, EGFR, ESR1, and PI3KCA. In addition, GO terms and KEGG pathway enrichment analyses revealed that Mingmu Dihuang pill may be effective for treatment of diabetic retinopathy through biological processes of positive regulation of cell migration, response to inorganic substance, transmembrane receptor protein tyrosine kinase signaling pathway via the AGE-RAGE signaling pathway in diabetic complications, PI3K-AKT signaling pathway, Rap1 signaling pathway, HIF-1 signaling pathway, VEGF signaling pathway, Erb B signaling pathway, and Foxo signaling pathway. It is therefore hypothesized that Mingmu Dihuang pill is active to mediate cell growth, participate in glucose metabolism, suppress cell apoptosis, and inhibit angiogenesis. Our data indicate that compound Chinese medicines function through multi-ingredient, multitarget, and multipathway actions. Molecular docking confirmed a good binding affinity of active ingredients of Mingmu Dihuang pill, including luteolin, acacetin, naringenin, and alisol B, with AKT1, SRC, and VEGFA, three hub targets of diabetic retinopathy. Of the four critical active ingredients of Mingmu Dihuang pill, luteolin, acacetin, and naringenin are only present in *C. morifolium*, and alisol B is only detected in *A. orientalis*.

The hub target AKT1 is highly expressed in insulin-sensitive tissues and triggers the downstream glucose transport protein to increase glucose uptake, thereby mediating glucose metabolism [25, 26]. The hub target SRC, a nonreceptor tyrosine kinase, plays a critical role in cell adhesion, cell cycle, and cell migration [27]. SRC was identified as a key gene associated with the risk of type 1 diabetes mellitus using a network biology approach [28], and inhibition of SRC activation resulted in a reduction in endogenous reactive oxygen species (ROS) production and an increase in ATP production in obese diabetic mouse models with hyperlipidemia [29]. In addition, selective SRC family kinase inhibition may serve as a novel attractive therapeutic intervention for retinal vascular pathology [30]. The hub target VEGFA is widely expressed in arterial, venous, and lymphatic microvessels and endothelial cells of large vessels [31], and VEGF inhibitors or anti-VEGR agents have been proven to be highly active against diabetic retinopathy [32, 33]. Our findings further demonstrate the feasibility and reliability of network pharmacology to investigate the mechanisms of drug actions.

Our findings showed that the three hub targets AKT1, VEGFA, and SRC were all significantly enriched in the Rap1 signaling pathway, and AKT1 and VEGFA were both significantly enriched in the AGE-RAGE signaling pathway in diabetic complications and PI3K-AKT signaling pathway. It is therefore hypothesized that Mingmu Dihuang pill may be active for treatment of diabetic retinopathy via these three signaling pathways. As a small GTPase, Rap1 is involved in multiple biological processes, including cell proliferation, migration, and adhesion [34]. Angiotensin-(1–7) is reported to suppress the acylation of the retinal protein O-GlcNAc via EPAC/Rap1-dependent O-GlcNAc transferase, suggesting the contribution of Rap1 to diabetic retinopathy [35]. Previous studies have shown that targeting Rap1A and inhibiting T cells infiltration alleviate diabetic peripheral neuropathic pain in mice [36], and Rap1B may prevent excessive vascular leakage in patients with early diabetes through suppressing VEGF signaling [37]. Advanced glycation end product-receptor for advanced glycation end product (AGE-RAGE) signaling, which is central to the pathogenesis of diabetes-associated complications, is involved in the injury of multiple types of cells, and this signaling is activated prior to development of apparent diabetic retinopathy [38]. AGEs are found to increase the permeability of retinal vascular endothelial cells through mediating chemokines or upregulating activator protein expression [39]. RAGE recognizes AGEs on cell membrane, and its downstream signals may activate the NF-*κ*B signaling pathway and increase the VEGF release, thereby leading to intracellular oxidative stress and inflammation [40]. Activation of the AGE-RAGE signaling induces Müller cell activation, upregulates VEGF expression, and promotes vascularization, thereby resulting in retinal vascular endothelial injury [41]. PI3K/AKT signaling is a major pathway in insulin, and aberrant PI3K/AKT signaling is a common pathogenesis of diabetes [42]. Previous studies have demonstrated that PI3K/AKT signaling plays a critical role in the neovascularization of diabetic retinopathy, and upon activation, PI3K/AKT signaling may extend the survival period of endothelial cells and synergize with VEGF to jointly mediate cell survival and migration, which finally triggers neovascularization [43, 44]. In addition, PI3K signaling may be activated by hypoglycemia and hypoxia under a diabetic condition, and activation of AKT may induce vascular dilation, remodeling, and vascularization [45]. Following activation, AKT may mediate multiple downstream target proteins, such as mediating cell growth and proliferation through phosphorylation of mTOR and mediating cell apoptosis through suppressing bad expression [46]. It has been found that activation of the PI3K/AKT signaling is involved in the pathophysiology of diabetic retinopathy and promotes the proliferation and migration of retinal endothelial cells [47]. These data demonstrate that the targets of Mingmu Dihuang pill actions identified by network pharmacology for diabetic retinopathy are in agreement with previous reports.

In summary, the results of the present study demonstrate that Mingmu Dihuang pill may be effective for the clinical treatment of diabetic retinopathy through active ingredients luteolin, acacetin, naringenin, and alisol B via AKT1, SRC, and VEGFA in AGE-RAGE, PI3K-AKT, and Rap1 signaling pathways. Development of novel agents from *C. morifolium* for treatment of diabetic retinopathy deserves further investigations. Our data provide insights into development of novel targets for the treatment of diabetic retinopathy.

## Figures and Tables

**Table 1:** GO terms enrichment analysis of 203 predicted targets.

Category	Term	Description	Log (*q* value)	Gene name
GO biological processes	GO:0030335	Positive regulation of cell migration	-41.57	AKT1, ALOX12, APP, SCARB1, MAPK14, EGFR, F3, FGF2, FGFR1, FLT1, MTOR, HIF1A, HMOX1, HRAS, ICAM1, IGF1R, IL6, CXCL8, INSR, ITGA2B, ITGA4, ITGB3, JAK2, KDR, LGALS3, MET, MMP9, MMP14, NOS3, SERPINE1, PDGFRB, PGF, PIK3CB, PIK3CD, PIK3CG, PIK3R1, PRKCA, MAPK1, MAPK3, PTGS2, PTPRC, RET, SELP, SRC, TEK, TERT, TGFBR2, TLR4, TNF, VEGFA, PLA2G7, AKT3, SIRT1, NOX4, CCR2, AGTR1, ALOX5, EDNRA, EPAS1, EPHB4, HK2, JUN, MMP2, PIK3CA, PPARG, PRKCB, SHH, EIF2AK3, AHR, APOB, LDLR, AR, CCND1, ERN1, ESR1, MEN1, VDR, and AGTR2
GO biological processes	GO:0010035	Response to inorganic substance	-37.67	ADA, PARP1, AKT1, AKR1B1, APOB, APP, CCND1, BRAF, CA2, CASP3, CASP9, CDK4, CYP1A2, DNMT3A, DRD2, EGFR, ERN1, FABP4, FABP1, G6PD, HIF1A, HMOX1, ICAM1, IGFBP2, IL6, JUN, KDR, MAPT, MET, MMP3, MMP9, MPO, MTTP, NOS3, NTRK1, PDGFRB, PIK3CA, PON1, PRKCD, MAPK1, MAPK3, MAPK8, MAPK10, PTGS2, SHH, SLC5A1, SORD, SRC, TERT, VCAM1, EIF2AK3, and SIRT1
GO biological processes	GO:1901652	Response to peptide	-32.80	PARP1, ADRB2, AGTR1, AGTR2, AKT1, AKR1B1, APP, CA2, CDK4, MAPK14, MTOR, GCGR, GCK, HSD11B2, ICAM1, IGF1R, IGFBP1, INSR, ITGA4, JAK2, KCNQ1, MC4R, MEN1, MMP2, MMP3, MMP9, NTRK1, PIK3CA, PIK3R1, PPARA, PPARG, PRKCB, PRKCD, PTGS2, PTPN1, PTPN2, RPS6KB1, SLC2A1, SRC, TEK, TLR4, TP53, VCAM1, TRPV1, NAMPT, SIRT1, ACHE, ADCY5, AHR, CASP3, CFTR, DNMT3A, DRD2, PIK3CG, MAPK1, MAPK3, TNF, NOX4, APOB, AR, CASP9, EGFR, ESR1, NR3C1, IGFBP2, NR3C2, PGF, and REN
GO biological processes	GO:0007169	Transmembrane receptor protein tyrosine kinase signaling pathway	-32.25	ADRB2, AGTR2, AKT1, AR, BLK, BRAF, CASP3, CDK4, MAPK14, EGFR, EPHB4, F3, FGF2, FGFR1, FLT1, HIF1A, IGF1R, IGFBP1, IGFBP2, IGFBP3, INSR, ITGB3, JAK2, KDR, MET, MMP2, MMP9, NTRK1, PDGFRB, PGF, PIK3CA, PIK3CB, PIK3CD, PIK3R1, PLAT, PRKCB, PRKCD, MAPK1, PTPN1, PTPN2, RET, RPS6KB1, SRC, TEK, VEGFA, EIF2AK3, NAMPT, MERTK, SIRT1, APP, CCND1, ACE, DRD2, ELANE, ERN1, F2, MTOR, HRAS, ICAM1, IL2, IL6, MIF, PIK3CG, PPARG, MAPK3, PTGS2, PTPRC, TGFBR2, TLR4, TNF, TNFRSF1A, TP53, CHEK2, NOX4, TLR9, FABP4, HMGCR, MAPT, MEN1, NEK2, ADRB3, CCR5, MMP8, PRKCA, MAPK8, MAPK10, REN, SIRT3, SELP, and PPARA
GO biological processes	GO:0009991	Response to extracellular stimulus	-31.97	ACACB, ADA, ADRB2, AKT1, CCND1, CNR1, CPT1A, CYP27B1, DNMT3A, EGFR, MTOR, G6PD, GCGR, HMGCR, HMOX1, HSD11B2, ICAM1, IGFBP2, ITGA4, JUN, LDLR, MC4R, MPO, NTRK1, PON1, PPARA, PPARG, MAPK1, MAPK3, MAPK8, PTGS2, PTPRC, RPS6KB1, SLC2A1, SORD, SRC, TGFBR2, TP53, NR1H2, VCAM1, VDR, TRPV1, EIF2AK3, NAMPT, SIRT1, ALOX12, APP, CASP9, MAPK14, FGF2, HRAS, IGFBP1, MIF, SERPINE1, PDGFRB, PRKCD, MAPK10, TERT, AKT3, CHEK2, SIRT3, NOX4, CASP1, TLR4, and TNFRSF1A
GO biological processes	GO:0010942	Positive regulation of cell death	-30.47	PARP1, AGTR2, AKT1, ALOX12, BLK, CASP3, CASP9, CDK4, CNR1, CTSD, DNMT3A, MTOR, HMGCR, HMOX1, IGFBP3, IL6, ITGA4, ITGB2, JAK2, JUN, MAPT, MMP3, MMP9, COX2, NOS2, NTRK1, PDGFRB, PIK3CB, PIK3CD, PPARG, PRF1, PRKCD, MAPK8, PTGS2, PTPN2, PTPRC, RET, SRC, TLR4, TNF, TNFRSF1A, TP53, VDR, TRPV1, CHEK2, SIRT1, and NOX4
GO biological processes	GO:0003013	Circulatory system process	-29.98	ADA, ADRB2, ADRB3, AGTR1, AGTR2, AKT1, AR, AVPR2, CACNA1C, CD38, CNR1, ACE, DRD2, EDNRA, EGFR, EPAS1, MTOR, GCGR, HMGCR, HMOX1, HSD11B2, ICAM1, IL2, INSR, JAK2, KCNJ5, KCNQ1, MTNR1B, NOS2, NOS3, PIK3CA, PIK3CG, PPARA, PPARG, PTGS1, PTGS2, REN, SGK1, SLC2A1, SLC5A1, SRC, TEK, VEGFA, TRPV1, NAMPT, NOX4, CDK4, MAPT, PRKCD, RET, and AKT3
GO biological processes	GO:0050878	Regulation of body fluid levels	-29.57	ADA, AKR1B1, ALOX12, AVPR2, CCND1, BLK, CFTR, MAPK14, DRD2, EGFR, F2, F3, HIF1A, HK2, HNF4A, HSD11B2, IL6, ITGA2B, ITGB3, JAK2, MET, COX2, NOS3, SERPINE1, PIK3CA, PIK3CB, PIK3CG, PLAT, PLG, PRKCA, PRKCD, SELP, SHH, SRC, TLR4, NR1H2, VDR, VEGFA, MERTK, ALOX5, CASP3, SCARB1, FGF2, MTOR, HMGCR, HMOX1, HRAS, KDR, PDGFRB, PPARA, TGFBR2, TNF, NTRK1, and CCR2
GO biological processes	GO:0070482	Response to oxygen levels	-28.66	ADA, AKT1, CASP1, CASP3, CD38, CDK4, DNMT3A, DRD2, EDNRA, EPAS1, FABP1, MTOR, HIF1A, HK2, HMOX1, HSD11B2, ICAM1, MMP2, MMP14, NOS2, PDGFRB, PGF, PLAT, PPARA, PTGS2, SLC2A1, SRC, TEK, TERT, TGFBR2, TP53, VCAM1, VEGFA, VHL, NAMPT, SIRT1, and NOX4
GO biological processes	GO:0002237	Response to molecule of bacterial origin	-26.82	AKT1, APOB, CASP1, CASP3, CASP9, SCARB1, CDK4, CCR5, CNR1, MAPK14, CYP1A2, CYP27B1, ELANE, ICAM1, IL6, CXCL8, JAK2, JUN, MPO, NOS2, NOS3, SERPINE1, PRKCA, MAPK1, MAPK3, MAPK8, PTGS2, REN, SELE, SELP, SRC, TLR4, TNF, VCAM1, TLR9, NLRP3, F2, FABP4, PRKCD, TNFRSF1A, TP53, EIF2AK3, KCNJ11, PTPN2, TRPV1, SIRT1, F3, HIF1A, PPARG, PTPN1, PTPRC, NR1H2, and CCR2
GO biological processes	GO:0062197	Cellular response to chemical stress	-26.59	PARP1, AKT1, AKR1B1, ALOX5, CASP3, EGFR, EPAS1, ERN1, FABP1, G6PD, HIF1A, HMOX1, IL6, JAK2, JUN, MAPT, MET, MMP2, MMP3, MMP9, MPO, NOS3, PIK3CA, PRKCD, MAPK1, MAPK3, MAPK8, MAPK10, PTGS2, SLC2A1, SRC, TLR4, TP53, EIF2AK3, SIRT1, ADA, APP, CD38, MMP14, PDGFRB, PTGS1, FGF2, IL2, NEK2, PTPRC, and MEN1
GO biological processes	GO:0050727	Regulation of inflammatory response	-25.79	ADA, AGTR1, ALOX5, APP, CASP1, CNR1, MAPK14, EGFR, ELANE, ESR1, FABP4, IL2, IL6, JAK2, LDLR, MMP3, MMP8, MMP9, SERPINE1, PIK3CG, PPARA, PPARG, PRKCD, PTGS2, PTPN2, PTPRC, SELE, SRC, TEK, TLR4, TNF, TNFRSF1A, PLA2G7, TLR9, NLRP3, CCR2, DRD2, MAPK3, PTPN1, NR1H2, F2, F3, FGF2, FGFR1, CXCL8, KDR, MET, PDGFRB, PGF, PLG, PRKCA, MAPK1, VEGFA, AGTR2, HIF1A, HK1, HMOX1, HRAS, MIF, NOS2, PIK3CD, PIK3R1, EIF2AK3, SIRT1, and MERTK
GO biological processes	GO:0018108	Peptidyl-tyrosine phosphorylation	-25.12	APP, BLK, ACE, EGFR, EPHB4, FGFR1, FLT1, MTOR, ICAM1, IGF1R, IL2, IL6, INSR, ITGB2, ITGB3, JAK2, KDR, MET, MIF, NTRK1, PDGFRB, PRKCD, MAPK3, PTPN1, PTPN2, PTPRC, RET, SRC, TEK, TNF, TNFRSF1A, TP53, VEGFA, MERTK, NOX4, AKT1, ERN1, NEK2, EIF2AK3, and CHEK2
GO biological processes	GO:0010817	Regulation of hormone levels	-24.12	ADCY5, AGTR1, AGTR2, AKR1B1, ALOX5, BLK, SCARB1, CD38, CFTR, CNR1, CPT1A, CYP1A2, CYP2C9, CYP2D6, CYP3A4, CYP19A1, CYP27B1, ACE, DRD2, EGFR, ESR1, GCK, HIF1A, HMGCR, HNF4A, IL6, JAK2, KCNJ11, KCNQ1, MTNR1B, NOS2, RBP4, REN, SHH, TNF, TTR, SIRT3, AKR1B10, ACHE, MAPK14, CTSD, HRAS, ITGB2, MAPT, PIK3R1, PRKCD, MAPK1, MAPK8, PTGS2, PTPN1, SRC, TLR4, NR1H2, ADA, AVPR2, HMOX1, PRKCB, SGK1, TNFRSF1A, TRPV1, CCR2, GCGR, MTTP, PIK3CD, PIK3CG, MERTK, and CA2
GO biological processes	GO:0097190	Apoptotic signaling pathway	-24.10	PARP1, AGTR2, AKT1, AR, CASP1, CASP3, CASP9, CD38, ERN1, FGFR1, HIF1A, HMOX1, HRAS, ICAM1, IL2, JAK2, JUN, LGALS3, MIF, MMP9, NOS3, SERPINE1, PIK3R1, PRKCA, PRKCD, MAPK8, PTGS2, PTPN1, PTPN2, PTPRC, RET, SHH, SRC, TERT, TNF, TNFRSF1A, TP53, EIF2AK3, CHEK2, SIRT1, APP, CCND1, MAPK14, DNMT3A, DRD2, EGFR, ELANE, MTOR, HMGCR, MEN1, MMP1, MMP2, MMP3, NTRK1, MAPK10, VCAM1, NAMPT, NOX4, AKR1B1, PIK3CA, MAPK3, SLC2A1, TLR4, TRPV1, ALOX5, IGF1R, MAPT, MET, MMP8, MAPK1, NR1H2, VEGFA, AKT3, and TLR9
GO biological processes	GO:0050900	Leukocyte migration	-24.10	ADA, AKT1, ALOX5, APP, CCR5, ELANE, FLT1, HMOX1, ICAM1, IL6, CXCL8, ITGA2B, ITGA4, ITGB2, LGALS3, MIF, MMP9, MMP14, SERPINE1, PGF, PIK3CD, PIK3CG, MAPK1, MAPK3, RET, SELE, SELP, SRC, TNF, VCAM1, VEGFA, PLA2G7, NLRP3, CCR2, AGTR1, MAPK14, EPHB4, F3, FGF2, FGFR1, GLI2, HRAS, ITGB3, KDR, MET, NTRK1, PDGFRB, PIK3CB, PRKCD, PTPN2, and SHH
GO biological processes	GO:0030155	Regulation of cell adhesion	-24.01	ADA, AKT1, ALOX12, ALOX5, BLK, CASP3, MAPK14, ELANE, GLI2, ICAM1, IGFBP2, IL2, IL6, CXCL8, ITGA4, ITGB2, JAK2, KDR, LGALS3, MEN1, MMP14, SERPINE1, PIK3CA, PIK3CB, PIK3CG, PIK3R1, PLG, PPARA, PRKCA, PRKCD, PTPN2, PTPRC, RET, SELE, SELP, SHH, SRC, TEK, TGFBR2, TNF, VCAM1, VEGFA, NLRP3, CCR2, PARP1, APP, CA2, FASN, MTOR, GLO1, JUN, MMP9, NTRK1, PIK3CD, PPARG, TLR4, TP53, MERTK, SIRT1, TLR9, AHR, CD38, CNR1, F2, HMOX1, LDLR, MIF, MMP8, NOS3, PDGFRB, PRF1, PRKCB, ACE, HK1, MPO, NOS2, RBP4, MAPK1, MAPK3, HIF1A, HRAS, and APOB
GO biological processes	GO:0071396	Cellular response to lipid	-23.27	ADCY5, PARP1, AGTR2, AHR, AKT1, ALOX12, AR, CASP1, CASP9, SCARB1, CDK4, CFTR, CCR5, CPT1A, MAPK14, CYP27B1, EGFR, ESR1, NR3C1, ICAM1, IL6, CXCL8, JAK2, LDLR, NR3C2, NOS2, SERPINE1, PPARA, PRKCA, MAPK1, MAPK3, MAPK8, RET, SRC, TLR4, TNF, VDR, SIRT1, NLRP3, APP, CASP3, DRD2, GLI2, KCNQ1, PIK3CG, PTGS2, TRPV1, CHEK2, NOX4, CCND1, CA2, CD38, HSD11B2, IGFBP2, TGFBR2, AKR1B1, and KCNJ11
GO biological processes	GO:0048661	Positive regulation of smooth muscle cell proliferation	-23.04	AKT1, AKR1B1, ALOX12, EGFR, ELANE, ERN1, FGF2, MTOR, HMGCR, HMOX1, IL6, JAK2, JUN, MMP2, MMP9, PDGFRB, PTGS2, TERT, TGFBR2, TLR4, TNF, NAMPT, MAPK14, IGFBP3, PPARG, MAPK1, RBP4, and SHH
GO biological processes	GO:0048871	Multicellular organismal homeostasis	-22.95	ACACB, ACHE, ADRB2, ADRB3, AKR1B1, ALOX12, CA2, CD38, CFTR, CNR1, CPT2, DRD2, EGFR, EPAS1, FABP4, IGF1R, IL6, ITGB3, JAK2, MC4R, MET, NOS3, PIK3CA, PRKCA, PTGS2, RBP4, SCD, SLC2A1, SRC, TLR4, TNF, NR1H2, VEGFA, TRPV1, AKT3, TLR9, CCR2, AGTR2, ACE, HIF1A, IL2, MMP2, MMP14, PLG, TP53, and VDR
GO cellular components	GO:0045121	Membrane raft	-19.04	APP, CASP3, SCARB1, CNR1, CTSD, EGFR, HK1, HMOX1, ICAM1, INSR, ITGB2, JAK2, KCNQ1, KDR, MAPT, NOS3, MAPK1, MAPK3, PTGS2, PTPRC, RET, SELE, SHH, SLC2A1, SRC, TEK, TGFBR2, TNF, and TNFRSF1A
GO cellular components	GO:0043235	Receptor complex	-14.82	ADRB2, ADRB3, AHR, APP, EGFR, EPHB4, FGFR1, FLT1, IGF1R, IL6, INSR, ITGA2B, ITGA4, ITGB2, ITGB3, KDR, LDLR, MET, NR3C2, MTTP, NTRK1, PDGFRB, PPARG, RET, TEK, TGFBR2, TLR4, TNFRSF1A, VDR, and MERTK
GO cellular components	GO:0098552	Side of membrane	-11.10	ACP1, ADA, AKT1, BLK, CA4, CCR5, ACE, F2, F3, G6PD, ICAM1, INSR, ITGA2B, ITGB2, KCNJ5, LDLR, PLG, PTPN1, PTPRC, SELE, SELP, SRC, TGFBR2, TLR4, TNF, VCAM1, TRPV1, and CCR2
GO cellular components	GO:0045177	Apical part of cell	-9.49	ADRB2, APP, CA2, CA4, CFTR, EGFR, FABP1, IGFBP2, KCNQ1, LDLR, PDGFRB, PLAT, REN, SI, SLC2A1, SLC2A2, SLC5A1, TEK, VCAM1, MGAM, NOX4, TLR9, CD38, MET, and MTTP
GO cellular components	GO:0031983	Vesicle lumen	-8.16	ADA, ALOX5, APOB, APP, MAPK14, CTSD, EGFR, ELANE, IMPDH1, MIF, MMP8, MPO, SERPINE1, PLG, PRKCD, MAPK1, TTR, VEGFA, ADRB2, AKR1B1, SCARB1, CFTR, ACE, MTOR, INSR, KCNQ1, LDLR, PDGFRB, PRF1, SRC, TLR9, and AKR1B10
GO cellular components	GO:0048471	Perinuclear region of cytoplasm	-6.18	ACHE, AGTR2, AKR1B1, ALOX5, APP, AVPR2, CA4, CDK4, EGFR, HMOX1, HRAS, NOS2, PIK3CA, PIK3R1, PRKCA, PRKCD, SELE, SLC5A1, SRC, TLR4, EIF2AK3, NOX4, and CCR2
GO cellular components	GO:0005769	Early endosome	-6.07	ADRB2, APOB, APP, CFTR, EGFR, KCNQ1, KDR, LDLR, NTRK1, MAPK1, MAPK3, PTPN1, RET, SLC5A1, TLR4, VCAM1, AOC3, AVPR2, SCARB1, DRD2, ELANE, MTOR, MPO, NOS3, TLR9, INSR, SRC, and CTSD
GO cellular components	GO:0043025	Neuronal cell body	-5.40	ADA, APOB, APP, CACNA1C, CASP3, DRD2, MTOR, INSR, ITGA4, KCNJ11, KCNQ1, MAPT, NTRK1, MAPK1, MAPK10, RET, TRPV1, CCR2, CA2, CNR1, HIF1A, IGF1R, PRKCB, MAPK8, AKT1, ITGB3, JAK2, and SRC
GO cellular components	GO:0031012	Extracellular matrix	-5.39	ACHE, CTSD, ELANE, F2, F3, ICAM1, LGALS3, MMP1, MMP2, MMP3, MMP8, MMP9, MMP14, SERPINE1, PLAT, PLG, PRSS1, SHH, and VEGFA
GO cellular components	GO:0005925	Focal adhesion	-5.10	EGFR, FLT1, ICAM1, ITGA2B, ITGA4, ITGB2, ITGB3, JAK2, MMP14, PDGFRB, MAPK1, MAPK3, PTPRC, SRC, TEK, NOX4, CACNA1C, CASP3, IGF1R, IL6, INSR, KCNJ1, KCNJ5, KCNJ11, KCNQ1, PRKCA, RET, VCAM1, and SELP
GO cellular components	GO:0005902	Microvillus	-4.57	AKR1B1, CA2, SCARB1, ITGB3, MTTP, TEK, VCAM1, AOC3, and APP
GO cellular components	GO:0005943	Phosphatidylinositol 3-kinase complex, class IA	-4.53	PIK3CA, PIK3CG, PIK3R1, PIK3CB, PIK3CD, AKT1, BLK, PLG, and SRC
GO cellular components	GO:0101002	Ficolin-1-rich granule	-4.12	ALOX5, MAPK14, CTSD, IMPDH1, ITGB2, LGALS3, MIF, MMP9, MAPK1, MGAM, and MMP8
GO cellular components	GO:0061695	Transferase complex, transferring phosphorus-containing groups	-4.03	CCND1, CDK4, ERN1, IGF1R, INSR, PIK3CA, PIK3CB, PIK3CD, PIK3CG, PIK3R1, TERT, and MEN1
GO cellular components	GO:0005788	Endoplasmic reticulum lumen	-3.72	APOB, APP, F2, IGFBP1, IGFBP3, IL6, MEN1, MTTP, MAPK1, MAPK3, PTGS2, and SHH
GO cellular components	GO:0005911	Cell-cell junction	-3.71	AKT1, AKR1B1, APP, CCND1, CDK4, ITGB3, KCNJ11, PIK3CA, PIK3R1, PRKCD, SLC2A1, SLC2A2, SLC5A1, TEK, and VEGFA
GO cellular components	GO:0031091	Platelet alpha granule	-3.62	APP, ITGA2B, ITGB3, SERPINE1, PLG, SELP, and VEGFA
GO cellular components	GO:0034358	Plasma lipoprotein particle	-3.55	APOB, CETP, LDLR, PON1, and PLA2G7
GO cellular components	GO:0031968	Organelle outer membrane	-3.48	ACACB, CNR1, CPT1A, CYP27B1, MTOR, HK1, HK2, HMOX1, PTGS2, RPS6KB1, CPT2, LGALS3, COX2, PRKCA, PTPN1, SORD, and SRC
GO cellular components	GO:0097443	Sorting endosome	-3.48	KDR, LDLR, PTPN1, PRKCD, and TLR9
GO cellular components	GO:0016773	Phosphotransferase activity, alcohol group as acceptor	-24.541	AKT1, CCND1, BLK, BRAF, CDK4, MAPK14, EGFR, EPHB4, ERN1, FGFR1, FLT1, MTOR, GCK, HK1, HK2, IGF1R, INSR, JAK2, KDR, MET, NEK2, NTRK1, PDGFRB, PIK3CA, PIK3CB, PIK3CD, PIK3CG, PRKCA, PRKCB, PRKCD, MAPK1, MAPK3, MAPK8, MAPK10, RET, RPS6KB1, SGK1, SRC, TEK, TGFBR2, EIF2AK3, AKT3, MERTK, and CHEK2
GO molecular functions	GO:0019900	Kinase binding	-13.744	PARP1, AKT1, CCND1, CASP1, CASP9, MAPK14, ACE, EGFR, ESR1, MTOR, NR3C1, HIF1A, ITGB2, JAK2, KCNQ1, KIF11, MAPT, NTRK1, PDGFRB, PIK3R1, PLG, PRKCB, PRKCD, MAPK1, PTPN1, PTPN2, PTPRC, SLC2A1, SRC, TGFBR2, TP53, CHEK2, SIRT1, and NOX4
GO molecular functions	GO:0019199	Transmembrane receptor protein kinase activity	-13.577	BLK, EGFR, EPHB4, FGFR1, FLT1, IGF1R, INSR, JAK2, KDR, MET, NTRK1, PDGFRB, PRKCD, RET, SRC, TEK, TGFBR2, MERTK, IGFBP1, IGFBP2, IGFBP3, and ITGB3
GO molecular functions	GO:0020037	Heme binding	-11.563	CYP1A2, CYP2C19, CYP2C9, CYP2D6, CYP3A4, CYP19A1, CYP27B1, HMOX1, JAK2, MPO, NOS2, NOS3, PTGS1, PTGS2, SRC, NOX4, ALDH2, AKR1B1, ALOX12, ALOX5, FASN, G6PD, HMGCR, HSD11B1, HSD11B2, IMPDH1, COX2, SCD, SORD, VCAM1, AOC3, and AKR1B10
GO molecular functions	GO:0008134	Transcription factor binding	-10.270	PARP1, AHR, AR, CCND1, MAPK14, DNMT3A, EPAS1, ESR1, MTOR, GLI2, HIF1A, HNF4A, JUN, PIK3R1, PPARA, PPARG, PRKCB, PTPN2, SRC, TERT, TP53, NR1H2, VDR, VHL, SIRT1, NLRP3, NR3C1, NR3C2, EGFR, FABP1, MEN1, and MPO
GO molecular functions	GO:0019902	Phosphatase binding	-9.464	AKT1, MAPK14, EGFR, HMGCR, KCNQ1, LGALS3, MAPT, MET, NEK2, PIK3R1, PPARA, MAPK1, MAPK3, PTPN1, TP53, and EIF2AK3
GO molecular functions	GO:0043560	Insulin receptor substrate binding	-7.850	IGF1R, INSR, JAK2, PIK3CA, PIK3R1, PRKCD, AR, EGFR, GCGR, NR3C1, MC4R, SHBG, TTR, PDGFRB, and REN
GO molecular functions	GO:0005539	Glycosaminoglycan binding	-7.446	APOB, APP, ELANE, F2, FGF2, FGFR1, HK1, MPO, PGF, PTPRC, SELP, SHH, TGFBR2, VEGFA, NLRP3, ACACB, GSTM1, and TKT
GO molecular functions	GO:0031994	Insulin-like growth factor I binding	-7.346	IGF1R, IGFBP1, IGFBP2, IGFBP3, INSR, and ITGB3
GO molecular functions	GO:0017171	Serine hydrolase activity	-7.206	ACHE, ACE, ELANE, F2, F3, MMP1, MMP2, MMP3, MMP8, MMP9, MMP14, PLAT, PLG, PRSS1, CASP1, CASP3, CASP9, CTSD, REN, and SHH
GO molecular functions	GO:0070851	Growth factor receptor binding	-7.012	APP, ERN1, FGF2, IL2, IL6, ITGB3, JAK2, PDGFRB, PGF, SRC, VEGFA, TLR9, CASP3, CXCL8, MIF, NTRK1, PIK3R1, TGFBR2, TNF, CCR2, LGALS3, AGTR2, F2, SHH, TTR, and NAMPT
GO molecular functions	GO:0005178	Integrin binding	-6.955	EGFR, FGF2, ICAM1, ITGA4, ITGB2, ITGB3, KDR, MMP14, PRKCA, PTPN2, SRC, VCAM1, FASN, and PTPN1
GO molecular functions	GO:0004712	Protein serine/threonine/tyrosine kinase activity	-6.852	AKT1, BRAF, MAPK14, MAPK1, MAPK3, MAPK10, RPS6KB1, SGK1, MAPK8, MTOR, MEN1, PIK3R1, PLAT, SRC, and TRPV1
GO molecular functions	GO:0005496	Steroid binding	-6.384	AR, CETP, CYP3A4, ESR1, NR3C1, HSD11B1, HSD11B2, NR3C2, SHBG, and VDR
GO molecular functions	GO:0042803	Protein homodimerization activity	-5.530	ACHE, ADRB2, ADRB3, AHR, AKT1, ERN1, FGFR1, G6PD, GSTM1, HMOX1, HNF4A, HSD11B1, NOS2, NTRK1, PON1, PTGS2, TERT, TKT, VEGFA, CHEK2, and TLR9
GO molecular functions	GO:0051879	Hsp90 protein binding	-5.503	AHR, ERN1, NR3C1, HIF1A, KDR, MAPT, EIF2AK3, ITGB2, KCNJ11, and PPARG
GO molecular functions	GO:0033218	Amide binding	-5.214	ACACB, ACHE, ADRB2, AVPR2, SCARB1, FASN, GCGR, GSTM1, IGF1R, INSR, ITGB2, LDLR, MC4R, PIK3R1, PPARG, and TLR4
GO molecular functions	GO:0035005	1-phosphatidylinositol-4-phosphate 3-kinase activity	-5.050	PIK3CA, PIK3CB, PIK3CD, and PIK3CG
GO molecular functions	GO:0031406	Carboxylic acid binding	-4.940	ACACB, FABP4, FABP1, FABP2, HNF4A, NOS2, NOS3, PPARG, SELE, SELP, VDR, and NR1H2
GO molecular functions	GO:0055056	D-glucose transmembrane transporter activity	-4.579	SLC2A1, SLC2A2, SLC5A1, SLC5A2, and RBP4

**Table 2:** KEGG pathway enrichment analysis of predicted target genes.

Term	Description	Log (*q* value)	Gene name
hsa05200	Pathways in cancer	-45.35	ADCY5, AGTR1, AKT1, AR, CCND1, BRAF, CASP3, CASP9, CDK4, EDNRA, EGFR, EPAS1, FGF2, FGFR1, MTOR, GLI2, HIF1A, HRAS, IGF1R, IL6, CXCL8, ITGA2B, JUN, MET, MMP1, MMP2, MMP9, NOS2, NTRK1, PDGFRB, PGF, PIK3CA, PIK3CB, PIK3CD, PIK3R1, PPARG, PRKCA, PRKCB, MAPK1, MAPK3, MAPK8, MAPK10, PTGS2, RET, SHH, SLC2A1, TGFBR2, TP53, VEGFA, VHL, AKT3, MAPK14, F3, ICAM1, JAK2, NOS3, SERPINE1, PRKCD, SELE, TNF, VCAM1, NOX4, ESR1, FASN, ITGB3, KDR, RPS6KB1, SRC, TLR4, FLT1, HK1, HK2, HMOX1, INSR, TEK, G6PD, GCK, SLC2A2, SIRT3, CYP2D6, IL2, ITGA4, PIK3CG, SGK1, CNR1, DRD2, ITGB2, GSTM1, PLAT, TNFRSF1A, ACE, TLR9, CASP1, PLG, PRSS1, EIF2AK3, NLRP3, SCARB1, LDLR, PPARA, MMP3, MMP14, CACNA1C, KCNJ11, PARP1, CTSD, ERN1, PRF1, KCNJ5, SIRT1, ACACB, PTPN1, CFTR, CPT1A, HMGCR, HNF4A, SCD, COX2, PTGS1, TERT, CHEK2, AVPR2, F2, MAPT, HSD11B2, KCNJ1, NR3C2, ALOX5, SI, SLC5A1, MGAM, ADRB2, ADRB3, FABP4, ACHE, KCNQ1, PTPRC, ALOX12, TRPV1, CCR5, CCR2, CD38, PTPN2, and EPHB4
hsa04931	Insulin resistance	-26.36	ACACB, AKT1, CPT1A, MTOR, IL6, INSR, NOS3, PIK3CA, PIK3CB, PIK3CD, PIK3R1, PPARA, PRKCB, PRKCD, MAPK8, MAPK10, PTPN1, RPS6KB1, SLC2A1, SLC2A2, TNF, TNFRSF1A, NR1H2, AKT3, JAK2, GCGR, GCK, PRKCA, and SIRT1
hsa05219	Bladder cancer	-18.69	CCND1, BRAF, CDK4, EGFR, HRAS, CXCL8, MMP1, MMP2, MMP9, MAPK1, MAPK3, SRC, TP53, VEGFA, NTRK1, PPARG, RET, CASP3, DNMT3A, MTOR, HMOX1, ITGB3, MET, PDGFRB, PIK3CA, PRKCA, PRKCB, PTGS2, and SIRT1
ko05152	Tuberculosis	-17.81	AKT1, CASP3, CASP9, MAPK14, CTSD, CYP27B1, IL6, ITGB2, JAK2, NOS2, MAPK1, MAPK3, MAPK8, MAPK10, SRC, TLR4, TNF, TNFRSF1A, VDR, AKT3, TLR9, ALOX5, CCR5, LDLR, PIK3CG, CASP1, CXCL8, JUN, NLRP3, MMP1, MMP3, MMP9, PTGS2, ITGA4, PRKCB, AHR, MTOR, HIF1A, IL2, TGFBR2, PRKCD, NAMPT, EGFR, and MET
hsa04726	Serotonergic synapse	-17.25	ADCY5, ALOX12, ALOX5, APP, BRAF, CACNA1C, CASP3, CYP2C19, CYP2C9, CYP2D6, HRAS, KCNJ5, PRKCA, PRKCB, MAPK1, MAPK3, PTGS1, and PTGS2
hsa04912	GnRH signaling pathway	-16.35	ADCY5, CACNA1C, MAPK14, EGFR, HRAS, JUN, MMP2, MMP14, PRKCA, PRKCB, PRKCD, MAPK1, MAPK3, MAPK8, MAPK10, SRC, CCND1, CD38, KCNJ5, NOS3, PIK3CG, PTGS2, CNR1, DRD2, PDGFRB, AKT1, AKT3, AGTR1, BRAF, EDNRA, IGF1R, GCK, KCNJ11, SLC2A1, SLC2A2, MTNR1B, SCARB1, LDLR, SIRT1, PDE11A, and TTR
hsa04371	Apelin signaling pathway	-13.17	ADCY5, AGTR1, AKT1, CCND1, MTOR, HRAS, NOS2, NOS3, SERPINE1, PIK3CG, PLAT, PRKCA, MAPK1, MAPK3, RPS6KB1, AKT3, ADRB2, ADRB3, CACNA1C, EDNRA, INSR, PDE5A, AGTR2, MAPK14, KCNQ1, ACE, REN, CD38, EGFR, PDGFRB, and PRKCB
ko05144	Malaria	-12.56	ICAM1, IL6, CXCL8, ITGB2, MET, SELE, SELP, TLR4, TNF, VCAM1, TLR9, FLT1, JUN, MMP1, MMP3, TEK, VEGFA, FASN, PRKCA, PRKCB, PARP1, PTGS2, and TNFRSF1A
ko05202	Transcriptional misregulation in cancer	-11.80	ELANE, FLT1, IGF1R, IGFBP3, IL6, CXCL8, MEN1, MET, MMP3, MMP9, MPO, NTRK1, PLAT, PPARG, TGFBR2, and TP53
hsa04520	Adherens junction	-10.69	ACP1, EGFR, FGFR1, IGF1R, INSR, MET, MAPK1, MAPK3, PTPN1, SRC, TGFBR2, BRAF, RPS6KB1, TNF, IL6, ADCY5, and AR
hsa04976	Bile secretion	-9.14	ADCY5, CA2, SCARB1, CFTR, CYP3A4, HMGCR, LDLR, PRKCA, SLC2A1, SLC5A1, CD38, KCNQ1, PRKCB, PRSS1, and KCNJ1
hsa04080	Neuroactive ligand-receptor interaction	-9.05	ADRB2, ADRB3, AGTR1, AGTR2, AVPR2, CNR1, DRD2, EDNRA, F2, GCGR, NR3C1, MC4R, MTNR1B, PLG, PRSS1, and TRPV1
hsa04913	Ovarian steroidogenesis	-9.05	ADCY5, ALOX5, SCARB1, CYP19A1, IGF1R, INSR, LDLR, PRKCA, and PTGS2
hsa00052	Galactose metabolism	-8.08	AKR1B1, GCK, HK1, HK2, SI, MGAM, AKR1B10, ALDH2, G6PD, and TKT
hsa03320	PPAR signaling pathway	-8.00	CPT1A, CPT2, FABP4, FABP1, FABP2, MMP1, PPARA, PPARG, SCD, FASN, and ALDH2
hsa05010	Alzheimer's disease	-7.75	APP, CACNA1C, CASP3, CASP9, ERN1, MAPT, COX2, MAPK1, MAPK3, TNF, TNFRSF1A, EIF2AK3, CASP1, IL6, CXCL8, ITGB2, TLR4, MAPK14, TP53, JAK2, JUN, MAPK8, MAPK10, and TLR9
hsa04144	Endocytosis	-7.57	ADRB2, ADRB3, CCR5, EGFR, FLT1, HRAS, IGF1R, KDR, LDLR, MET, NTRK1, RET, SRC, TGFBR2, IL2, IL6, CXCL8, PDGFRB, TNF, TNFRSF1A, VEGFA, and CCR2
ko04137	Mitophagy-animal	-7.09	HIF1A, HRAS, JUN, MAPK8, MAPK10, SRC, TP53, EIF2AK3, CCND1, PRKCA, PRKCB, CDK4, and CFTR
hsa04115	p53 signaling pathway	-6.89	CCND1, CASP3, CASP9, CDK4, IGFBP3, SERPINE1, TP53, CHEK2, ICAM1, ITGB2, and PRF1
hsanan01	Drug metabolism	-6.20	CYP1A2, CYP2C19, CYP2C9, CYP2D6, CYP3A4, GSTM1, IMPDH1, MPO, SORD, HSD11B1, PTGS2, CYP19A1, and HSD11B2

**Table 3:** KEGG pathway enrichment analysis of potential targets of Mingmu Dihuang pill for treatment of diabetic retinopathy.

No.	Signaling pathway	Frequency
1	Pathways in cancer	51
2	AGE-RAGE signaling pathway in diabetic complications	34
3	Proteoglycans in cancer	36
4	HIF-1 signaling pathway	30
5	Endocrine resistance	27
6	Central carbon metabolism in cancer	25
7	PI3K-Akt signaling pathway	39
8	Rap1 signaling pathway	33
9	EGFR tyrosine kinase inhibitor resistance	24
10	Insulin resistance	24
11	TNF signaling pathway	24
12	Focal adhesion	28
13	Type II diabetes mellitus	18
14	VEGF signaling pathway	18
15	Apoptosis	23
16	Ras signaling pathway	27
17	Erb B signaling pathway	19
18	Prolactin signaling pathway	18
19	Foxo signaling pathway	21
20	Insulin signaling pathway	21

**Table 4:** Molecular docking of active ingredients of Mingmu Dihuang pill with key targets for diabetic retinopathy.

Target	Active ingredient	Docking energy (kcal/mol)
AKT1	Luteolin	–9.7
AKT1	Acacetin	–9.4
AKT1	Alisol B	–7.9
AKT1	Naringenin	–8.0
SRC	Luteolin	–8.4
SRC	Acacetin	–8.4
SRC	Alisol B	–9.0
SRC	Naringenin	–7.6
VEGFA	Luteolin	–8.1
VEGFA	Acacetin	–7.6
VEGFA	Alisol B	–8.3
VEGFA	Naringenin	–6.9

## Data Availability

All data presented in the study are available upon request by contact with the corresponding author.

## Supplementary Materials

Supplementary Materials The active ingredients of Mingmu Dihuang Pill are described in Supplementary Table 1.
