# Supplementary material for: Identification of Potential Molecular Targets and Active Ingredients of Mingmu Dihuang Pill for the Treatment of Diabetic Retinopathy Based on Network Pharmacology
Source: Biomed Res Int. 2022 Nov 24;2022:2896185. doi: 10.1155/2022/2896185 (PMC9715340; doi:10.1155/2022/2896185)
Supplement: Supplementary Materials — The active ingredients of Mingmu Dihuang Pill are described in Supplementary Table 1. [file 2896185.f1.docx]

**Supplementary Table 1. Active ingredients of Mingmu Dihuang Pill**

| Traditional Chinese medicine | ID | MOL ID | Main active ingredients | Oral bioavailability (%) | Drug likeness |
| --- | --- | --- | --- | --- | --- |
| *Chrysanthemum morifolium* | JH1 | MOL003044 | Chryseriol | 35.850895 | 0.27415 |
|  | JH2 | MOL011816 | [(1S,5S,7S)-7-acetoxy-5-isopropenyl-2,8-dimethylene-cyclodecyl] acetate | 37.024568 | 0.19315 |
|  | JH3 | MOL001689 | Acacetin | 34.973573 | 0.24082 |
|  | JH4 | MOL001790 | Linarin | 39.843731 | 0.70925 |
|  | JH5 | MOL005100 | 5,7-dihydroxy-2-(3-hydroxy-4-methoxyphenyl)chroman-4-one | 47.736437 | 0.27226 |
|  | JH6 | MOL000006 | Luteolin | 36.162629 | 0.24552 |
|  | JH7 | MOL001506 | Supraene | 33.545943 | 0.42161 |
|  | JH7 | MOL001733 | Eupatorin | 30.232217 | 0.37395 |
|  | JH8 | MOL001755 | 24-Ethylcholest-4-en-3-one | 36.083612 | 0.75703 |
|  | JH9 | MOL002881 | Diosmetin | 31.137949 | 0.27442 |
|  | JH10 | MOL004328 | Naringenin | 59.293898 | 0.21128 |
|  | JH11 | MOL005229 | Artemetin | 49.550723 | 0.4787 |
|  | JH12 | MOL007326 | Cynarin(e) | 31.758501 | 0.67849 |
|  | JH13 | MOL011319 | Truflex OBP | 43.735088 | 0.243 |
|  | JH14 | MOL011802 | (24r)-saringosterol | 39.359539 | 0.78836 |
| *Lycium barbarum* | GQ1 | MOL001323 | Sitosterol alpha1 | 43.28127 | 0.78354 |
|  | GQ2 | MOL003578 | Cycloartenol | 38.685659 | 0.78093 |
|  | GQ3 | MOL001979 | LAN | 42.119189 | 0.74787 |
|  | GQ4 | MOL005406 | atropine | 45.970582 | 0.19328 |
|  | GQ5 | MOL007449 | 24-methylidenelophenol | 44.192645 | 0.7533 |
|  | GQ6 | MOL008173 | daucosterol_qt | 36.913906 | 0.75316 |
|  | GQ7 | MOL008400 | glycitein | 50.478914 | 0.23826 |
|  | GQ8 | MOL009612 | (24R)-4alpha-Methyl-24-ethylcholesta-7,25-dien-3beta-ylacetate | 46.357499 | 0.8398 |
|  | GQ9 | MOL009615 | 24-Methylenecycloartan-3beta,21-diol | 37.317282 | 0.79751 |
|  | GQ10 | MOL009617 | 24-ethylcholest-22-enol | 37.094541 | 0.7511 |
|  | GQ11 | MOL009618 | 24-ethylcholesta-5,22-dienol | 43.829852 | 0.75636 |
|  | GQ12 | MOL009620 | 24-methyl-31-norlanost-9(11)-enol | 37.999685 | 0.75092 |
|  | GQ13 | MOL009621 | 24-methylenelanost-8-enol | 42.368199 | 0.76769 |
|  | GQ14 | MOL009622 | Fucosterol | 43.776396 | 0.75668 |
|  | GQ15 | MOL009631 | 31-Norcyclolaudenol | 38.682096 | 0.81391 |
|  | GQ16 | MOL009633 | 31-norlanost-9(11)-enol | 38.353941 | 0.7249 |
|  | GQ17 | MOL009634 | 31-norlanosterol | 42.204621 | 0.73012 |
|  | GQ18 | MOL009635 | 4,24-methyllophenol | 37.834674 | 0.74999 |
|  | GQ19 | MOL009639 | Lophenol | 38.129403 | 0.714 |
|  | GQ20 | MOL009640 | 4alpha,14alpha,24-trimethylcholesta-8,24-dienol | 38.90989 | 0.75772 |
|  | GQ21 | MOL009641 | 4alpha,24-dimethylcholesta-7,24-dienol | 42.653041 | 0.75297 |
|  | GQ22 | MOL009642 | 4alpha-methyl-24-ethylcholesta-7,24-dienol | 42.295095 | 0.78304 |
|  | GQ23 | MOL009644 | 6-Fluoroindole-7-Dehydrocholesterol | 43.726025 | 0.72224 |
|  | GQ24 | MOL009646 | 7-O-Methylluteolin-6-C-beta-glucoside_qt | 40.773688 | 0.30497 |
|  | GQ25 | MOL009650 | Atropine | 42.158971 | 0.19299 |
|  | GQ26 | MOL009651 | Cryptoxanthin monoepoxide | 46.953719 | 0.56103 |
|  | GQ27 | MOL009653 | Cycloeucalenol | 39.726472 | 0.79446 |
|  | GQ28 | MOL009656 | (E,E)-1-ethyl octadeca-3,13-dienoate | 41.9962 | 0.19364 |
|  | GQ29 | MOL009660 | methyl (1R,4aS,7R,7aS)-4a,7-dihydroxy-7-methyl-1-[(2S,3R,4S,5S,6R)-3,4,5-trihydroxy-6-(hydroxymethyl)oxan-2-yl]oxy-1,5,6,7a-tetrahydrocyclopenta[d]pyran-4-carboxylate | 39.428477 | 0.46558 |
|  | GQ30 | MOL009664 | Physalin A | 91.706475 | 0.27207 |
|  | GQ31 | MOL009665 | Physcion-8-O-beta-D-gentiobioside | 43.903587 | 0.62426 |
|  | GQ32 | MOL009677 | lanost-8-en-3beta-ol | 34.226304 | 0.74036 |
|  | GQ33 | MOL009678 | lanost-8-enol | 34.226304 | 0.74167 |
|  | GQ34 | MOL009681 | Obtusifoliol | 42.552002 | 0.7565 |
| *Moutan cortex* | MDP1 | MOL007003 | benzoyl paeoniflorin | 31.138666 | 0.54227 |
|  | MDP2 | MOL007374 | 5-[[5-(4-methoxyphenyl)-2-furyl]methylene]barbituric acid | 43.44402 | 0.30018 |
| *Dioscoreae rhizoma* | SY1 | MOL001559 | piperlonguminine | 30.711427 | 0.1802 |
|  | SY2 | MOL000310 | Denudatin B | 61.472376 | 0.37838 |
|  | SY3 | MOL000322 | Kadsurenone | 54.723013 | 0.37829 |
|  | SY4 | MOL005429 | hancinol | 64.013268 | 0.37314 |
|  | SY5 | MOL005435 | 24-Methylcholest-5-enyl-3belta-O-glucopyranoside_qt | 37.576818 | 0.71653 |
|  | SY6 | MOL005440 | Isofucosterol | 43.776396 | 0.7576 |
|  | SY7 | MOL005458 | Dioscoreside C_qt | 36.382287 | 0.87051 |
|  | SY8 | MOL000546 | diosgenin | 80.877925 | 0.80979 |
|  | SY9 | MOL005461 | Doradexanthin | 38.15575 | 0.53662 |
| *Poria cocos* | FL1 | MOL000273 | (2R)-2-[(3S,5R,10S,13R,14R,16R,17R)-3,16-dihydroxy-4,4,10,13,14-pentamethyl-2,3,5,6,12,15,16,17-octahydro-1H-cyclopenta[a]phenanthren-17-yl]-6-methylhept-5-enoic acid | 30.932142 | 0.81281 |
|  | FL2 | MOL000275 | trametenolic acid | 38.7115 | 0.80199 |
|  | FL3 | MOL000276 | 7,9(11)-dehydropachymic acid | 35.105891 | 0.81091 |
|  | FL4 | MOL000279 | Cerevisterol | 37.963828 | 0.77061 |
|  | FL5 | MOL000280 | (2R)-2-[(3S,5R,10S,13R,14R,16R,17R)-3,16-dihydroxy-4,4,10,13,14-pentamethyl-2,3,5,6,12,15,16,17-octahydro-1H-cyclopenta[a]phenanthren-17-yl]-5-isopropyl-hex-5-enoic acid | 31.072057 | 0.81528 |
|  | FL6 | MOL000282 | ergosta-7,22E-dien-3beta-ol | 43.507086 | 0.71939 |
|  | FL7 | MOL000283 | Ergosterol peroxide | 40.36268 | 0.81255 |
|  | FL8 | MOL000285 | (2R)-2-[(5R,10S,13R,14R,16R,17R)-16-hydroxy-3-keto-4,4,10,13,14-pentamethyl-1,2,5,6,12,15,16,17-octahydrocyclopenta[a]phenanthren-17-yl]-5-isopropyl-hex-5-enoic acid | 38.255158 | 0.82014 |
|  | FL9 | MOL000287 | 3beta-Hydroxy-24-methylene-8-lanostene-21-oic acid | 38.699914 | 0.8095 |
|  | FL10 | MOL000289 | pachymic acid | 33.62792 | 0.81076 |
|  | FL11 | MOL000290 | Poricoic acid A | 30.606946 | 0.76152 |
|  | FL12 | MOL000291 | Poricoic acid B | 30.524601 | 0.7463 |
|  | FL13 | MOL000292 | poricoic acid C | 38.151358 | 0.74643 |
|  | FL14 | MOL000296 | hederagenin | 36.913906 | 0.75072 |
|  | FL15 | MOL000300 | dehydroeburicoic acid | 44.172299 | 0.83458 |
| *Alisma orientalis* | ZX1 | MOL000830 | Alisol B | 34.473073 | 0.81706 |
|  | ZX2 | MOL000831 | Alisol B monoacetate | 35.576236 | 0.80629 |
|  | ZX3 | MOL000832 | alisol,b,23-acetate | 32.516216 | 0.81841 |
|  | ZX4 | MOL000849 | 16β-methoxyalisol B monoacetate | 32.427241 | 0.7679 |
|  | ZX5 | MOL000853 | alisol B | 36.760381 | 0.81993 |
|  | ZX6 | MOL000854 | alisol C | 32.700169 | 0.81507 |
|  | ZX7 | MOL000856 | alisol C monoacetate | 33.063589 | 0.82763 |
|  | ZX8 | MOL002464 | 1-Monolinolein | 37.176628 | 0.30249 |
|  | ZX9 | MOL000862 | [(1S,3R)-1-[(2R)-3,3-dimethyloxiran-2-yl]-3-[(5R,8S,9S,10S,11S,14R)-11-hydroxy-4,4,8,10,14-pentamethyl-3-oxo-1,2,5,6,7,9,11,12,15,16-decahydrocyclopenta[a]phenanthren-17-yl]butyl] acetate | 35.576236 | 0.80765 |
| *Cornus officinalis* | SZY1 | MOL002879 | Diop | 43.593325 | 0.39247 |
|  | SZY2 | MOL002883 | Ethyl oleate (NF) | 32.397388 | 0.19061 |
|  | SZY3 | MOL003137 | Leucanthoside | 32.115893 | 0.78146 |
|  | SZY4 | MOL005481 | 2,6,10,14,18-pentamethylicosa-2,6,10,14,18-pentaene | 33.404117 | 0.24028 |
|  | SZY5 | MOL005489 | 3,6-Digalloylglucose | 31.415212 | 0.66343 |
|  | SZY6 | MOL005503 | Cornudentanone | 39.663406 | 0.327 |
|  | SZY7 | MOL008457 | Tetrahydroalstonine | 32.419775 | 0.81311 |
|  | SZY8 | MOL000554 | Gallicacid-3-O-(6'-O-galloyl)-glucoside | 30.250322 | 0.6746 |
|  | SZY9 | MOL005557 | lanosta-8,24-dien-3-ol,3-acetate | 44.29554 | 0.82425 |
| *Paeonia lactiflora* | BS1 | MOL001910 | 11alpha,12alpha-epoxy-3beta-23-dihydroxy-30-norolean-20-en-28,12beta-olide | 64.773893 | 0.37586 |
|  | BS2 | MOL001919 | (3S,5R,8R,9R,10S,14S)-3,17-dihydroxy-4,4,8,10,14-pentamethyl-2,3,5,6,7,9-hexahydro-1H-cyclopenta[a]phenanthrene-15,16-dione | 43.556202 | 0.53276 |
|  | BS3 | MOL001924 | paeoniflorin | 53.870375 | 0.78709 |
|  | BS4 | MOL001921 | Lactiflorin | 49.121317 | 0.79711 |
| *Tribulus terrestris* | JL1 | MOL000483 | (Z)-3-(4-hydroxy-3-methoxy-phenyl)-N-[2-(4-hydroxyphenyl)ethyl]acrylamide | 118.34775 | 0.26399 |
|  | JL2 | MOL008563 | (3R,8S,9S,10R,13R,14R,17S)-17-((2S,5R)-5-ethyl-6-methylheptan-2-yl)-3-hydroxy-10,13-dimethyl-3,4,8,9,10,11,12,13,14,15,16,17-dodecahydro-1H-cyclopenta[a]phenanthren-7(2H)-one | 40.927008 | 0.79013 |
|  | JL3 | MOL008567 | (3R,7R,8S,9S,10S,13R,14S,17R)-17-((2R,5S)-5-ethyl-6-methylheptan-2-yl)-3,10-dimethyl-2,3,4,7,8,9,10,11,12,13,14,15,16,17-tetradecahydro-1H-cyclopenta[a]phenanthren-7-ol | 34.211333 | 0.76401 |
|  | JL4 | MOL008568 | (Z)-3-(3,4-dihydroxyphenyl)-N-[2-(4-hydroxyphenyl)ethyl]acrylamide | 113.25047 | 0.23596 |
|  | JL5 | MOL008588 | β-sitosterol-β-D-glucopyranoside | 32.413804 | 0.70881 |
|  | JL6 | MOL008590 | terrestriamide | 114.08801 | 0.29408 |
| *Concha haliotidis* | SJM1 | caffeine |  |  |  |
|  | SJM2 | conchiolin |  |  |  |
|  | SJM3 | dibutyl phthalate |  |  |  |
| Common* | A | MOL000354 | isorhamnetin | 49.604377 | 0.306 |
|  | B | MOL000422 | kaempferol | 41.88225 | 0.24066 |
|  | C1 | MOL000098 | quercetin | 46.433348 | 0.27525 |
|  | C2 | MOL000358 | beta-sitosterol | 36.913906 | 0.75123 |
|  | D | MOL001771 | poriferast-5-en-3beta-ol | 36.913906 | 0.75034 |
|  | E1 | MOL001494 | Mandenol | 41.9962 | 0.19321 |
|  | E2 | MOL001495 | Ethyl linolenate | 46.100963 | 0.19716 |
|  | E3 | MOL000449 | Stigmasterol | 43.829852 | 0.75665 |
|  | F1 | MOL005438 | campesterol | 37.576818 | 0.71488 |
|  | F2 | MOL000953 | CLR | 37.873898 | 0.67677 |
|  | G1 | MOL001925 | paeoniflorin_qt | 68.175762 | 0.39507 |
|  | G2 | MOL000211 | Mairin | 55.377073 | 0.7761 |
|  | G3 | MOL000359 | sitosterol | 36.913906 | 0.7512 |

* The common ingredients of A include *Chrysanthemum morifolium* and *Tribulus terrestris*; the common ingredients of B include *C. morifolium*, *Moutan cortex*, *Paeonia lactiflora* and *Tribulus terrestris*; the common ingredients of C1 include *C. morifolium*, *Lycium barbarum* and *M. cortex*; the common ingredients of C2 include *C. morifolium*, *L. barbarum*, *Radix rehmanniae preparata*, *Cornus officinalis*, *Angelica sinensis* and *P. lactiflora*; the common ingredients of D include *C. morifolium* and *C. officinalis*; the common ingredients of E1 include *L. barbarum* and *C. officinalis*; the common ingredients of E2 include *L. barbarum* and *C. officinalis*; the common ingredients of E3 include *L. barbarum*, *C. officinalis*, *R. rehmanniae preparata*, *Dioscoreae rhizoma* and *A. sinensis*; the common ingredients of F1 include *L. barbarum* and *Dioscoreae rhizoma*; the common ingredients of F2 include *L. barbarum* and *Dioscoreae rhizoma*; the common ingredients of G1 include *M. cortex* and *P. lactiflora*; the common ingredients of G2 include *M. cortex* and *P. lactiflora*; the common ingredients of G3 include *M. cortex*, *Alisma orientalis*, *C. officinalis*, *P. lactiflora* and *Tribulus terrestris*.
